# Reduced local input to fast‐spiking interneurons in the somatosensory cortex in the GABA_A_ γ2 R43Q mouse model of absence epilepsy

**DOI:** 10.1111/epi.13693

**Published:** 2017-02-13

**Authors:** Stephen P. Currie, Liliana L. Luz, Sam A. Booker, David J. A. Wyllie, Peter C. Kind, Michael I. Daw

**Affiliations:** ^1^ Muir Maxwell Epilepsy CentrePatrick Wild CentreCentre for Integrative PhysiologyUniversity of EdinburghEdinburghUnited Kingdom; ^2^ Centre for Brain Development and RepairInStemBangaloreIndia; ^3^ Present address: Institute for Molecular and Cell BiologyUniversity of PortoPortoPortugal

**Keywords:** Animal models, Inhibition, Synapse, Development

## Abstract

**Objective:**

Absence seizures in childhood absence epilepsy are initiated in the thalamocortical (TC) system. We investigated if these seizures result from altered development of the TC system before the appearance of seizures in mice containing a point mutation in γ‐aminobutyric acid A (GABA_A_) receptor γ2 subunits linked to childhood absence epilepsy (R43Q). Findings from conditional mutant mice indicate that expression of normal γ2 subunits during preseizure ages protect from later seizures. This indicates that altered development in the presence of the *R43Q* mutation is a key contributor to the R43Q phenotype. We sought to identify the cellular processes affected by the *R43Q* mutation during these preseizure ages.

**Methods:**

We examined landmarks of synaptic development at the end of the critical period for somatosensory TC plasticity using electrophysiologic recordings in TC brain slices from wild‐type mice and R43Q mice.

**Results:**

We found that the level of TC connectivity to layer 4 (L4) principal cells and the properties of TC synapses were unaltered in R43Q mice. Furthermore, we show that, although TC feedforward inhibition and the total level of GABAergic inhibition were normal, there was a reduction in the local connectivity to cortical interneurons. This reduction leads to altered inhibition during bursts of cortical activity.

**Significance:**

This altered inhibition demonstrates that alterations in cortical circuitry precede the onset of seizures by more than a week.

**Key Points**

- Normal thalamocortical input to principal cells and interneurons of somatosensory cortex during development in an R43Q absence epilepsy model
- Normal total inhibition and feedforward inhibition in response to single thalamic stimuli
- Reduced local connectivity to fast‐spiking interneurons and altered inhibition during bursts develop significantly before seizure onset

Childhood absence epilepsy is a common form of generalized epilepsy presenting early in life (4 to 10 years old) typified by frequent seizures in which unresponsiveness is coincident with spike‐wave discharges on electroencephalography (EEG).^1^ Studies in animal models of childhood absence epilepsy have consistently implicated the thalamocortical (TC) circuit in the generation of seizures,^2^, ^3^, ^4^, ^5^ with initiation occurring in the primary somatosensory cortex (S1).^4^ EEG and functional magnetic resonance imaging (fMRI) recordings in humans are broadly consistent with the TC hypothesis,^6^, ^7^ and also suggest that increased activity in parietal cortex (containing S1) precedes the onset of seizures.^8^ Thus the available evidence indicates that S1 cortical activity results in seizures propagated by the TC circuit.

A point mutation in the γ‐aminobutyric acid A (GABA_A_) receptor γ2 subunit (R43Q) has been identified in an Australian family with a history of childhood absence epilepsy and febrile seizures.^9^ Mice heterozygous for the same mutation in the γ2 subunit develop seizures, consisting of behavioral arrest and spike‐wave discharges, at a developmental stage (~postnatal day 22 [P22]) that parallels the developmental onset seen in patients.^10^

The γ2 subunit is distributed throughout the central nervous system and is near‐ubiquitous at synaptic GABA_A_ receptors.^11^, ^12^ As such, mutations in this receptor fundamentally alter the phasic inhibition mediated by synaptic GABA_A_ receptors. The *R43Q* mutation has been reported to affect a number of properties of GABA_A_ receptor function,^13^ but the most consistent finding is a reduction in surface expression of mutant receptors, reported in non‐neuronal cells,^14^, ^15^, ^16^, ^17^ cultured neurons,^14^, ^18^ and neurons in situ.^10^ Consistent with this reduction, the amplitude of miniature inhibitory postsynaptic currents (IPSCs) is reduced in layer 2/3 pyramidal cells of S1 in heterozygous mice aged P14–16.^10^ These changes appear to accurately reflect the situation in patients with the mutation, as they show a reduction in total benzodiazepine binding representing reduced surface GABA_A_ receptors^19^ and reduced intracortical inhibition as shown by transcranial magnetic stimulation.^20^

Intuitively this reduction in cortical inhibition could directly explain the appearance of seizures, but a conditional mutant mouse model demonstrates that acute expression of the mutant allele does not fully recapitulate the seizure disorder.^21^ Indeed, expression of wild‐type receptors throughout early development protected against full seizure development later in life. It is not known what developmental processes are affected by the *R43Q* mutation, but this period coincides with the critical period for somatosensory TC development^22^, ^23^ and the experience‐dependent increase in inhibition in S1.^24^, ^25^ Furthermore, disruption of normal neonatal GABAergic signaling has profound effects on the development of somatosensory cortex,^26^ and in the visual cortex, the development of inhibition is crucial for correct developmental synaptic plasticity.^27^ As such, reduced GABAergic signaling in early life in *R43Q* mutant mice could affect the development of multiple elements of the TC‐S1 circuit. Herein we examine the development of excitatory and feedforward inhibitory TC circuits following the critical period in the first postnatal week in somatosensory cortex. We find that although total GABAergic inhibition in cortical layer 4 (L4) is unaltered, the local input to interneurons is reduced in R43Q mice. This results in an altered pattern of inhibition in response to physiologically relevant trains of thalamic stimulation. These findings highlight the complex developmental consequences of epilepsy‐causing mutations.

## Materials and Methods

### Slice preparation

All animal experiments were approved by a University of Edinburgh internal ethics committee and were performed under license by the UK Home Office. TC slices were prepared from male and female P9 to P11 (P0 is designated as the day of birth) littermate wild‐type and heterozygous R43Q mice^10^ on a C57/Bl6 background (provided by Steven Petrou and Bionomics) as described previously.^25^, ^28^, ^29^ Brains were sliced in ice‐cold partial sucrose cutting solution: 80 mm NaCl, 2.5 mm KCl, 1.25 mm NaH_2_PO_4_, 25 mm NaHCO_3_, 10 mm glucose, 90 mm sucrose, 0.5 mm CaCl_2_, and 4.5 mm MgSO_4_ saturated with 95% O2/5% CO_2,_ pH 7.4_._ Slices were transferred in sucrose cutting solution to a water bath at 35°C for 30 min and then maintained at room temperature until recording.

### Recording conditions

**Figure 1:**
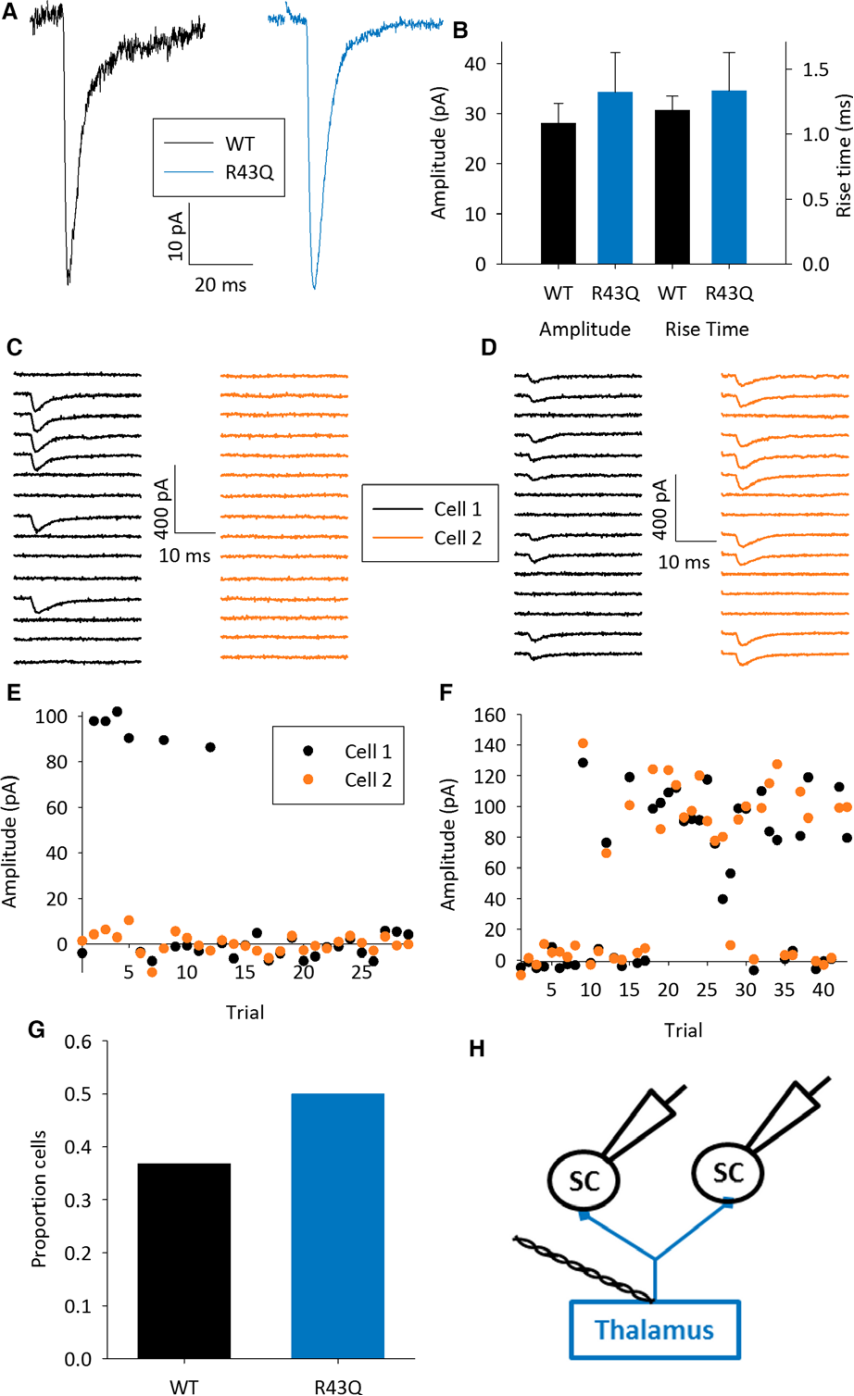
Normal TC connectivity in barrel cortex. (**A**) Example EPSCs evoked by minimal stimulation (excluding failures) in L4 SCs in WT (black) and R43Q (blue) mice. (**B**) Bar chart showing average msEPSC amplitude and rise time. Scale bars in this and subsequent figures show standard error of the mean (sem). (**C**) Fifteen consecutive responses to minimal thalamic stimulation in two simultaneously recorded L4 SCs showing EPSCs in cell 1 (black) but not cell 2 (orange). (**D**) Example traces from simultaneously recorded L4 SCs showing coincident EPSCs and failures in cell 1 and cell 2. (**E**) Amplitude versus trial number plot for cells shown in **C**. (**F**) Amplitude versus trial number plot for cells shown in **D**. (**G**). Bar graph showing proportion of experiments in which both cells showed coincident EPSCs and failures (as in **D**,** F**) for WT and R43Q mice. (**H**) Schematic of recording configuration SCs in black, and thalamus and TC axons in blue.

**Figure 2:**
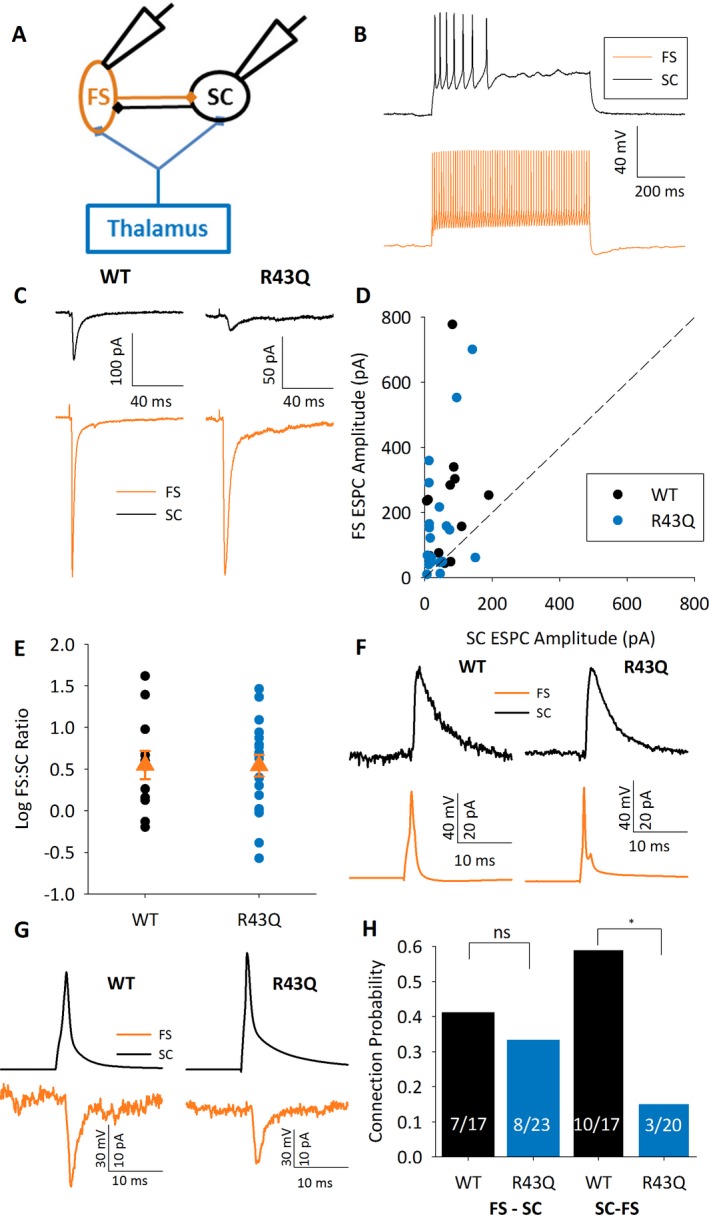
Reduced local input to FS interneurons. (**A**) Schematic of recording configuration SC in black, FS in orange, and thalamus and TC axons in blue. (**B**) Typical firing patterns displayed by SCs and FS cells to 500 msec depolarizing steps of double threshold amplitude. (**C**) Example traces showing EPSCs evoked by TC stimulation in simultaneously recorded SCs and FS cells in WT and R43Q mice. (**D**) Graph of mean EPSC amplitude in FS versus SC for individual experiments in WT and R43Q mice. (**E**) Log FS EPSC‐to‐SC EPSC ratio for experiments shown in **D**. (**F**) Example traces showing unitary IPSCs in SCs recorded at a holding potential of −30 mV evoked by stimulation of simultaneously recorded FS cells. (**G**) Example traces showing unitary EPSCs in FS cells recorded at a holding potential of −70 mV evoked by stimulation of simultaneously recorded SCs. (**H**) Bar graph showing FS to SC and SC to FS connection probability in all experiments. Numbers in bars represent n:pairs connected/n:pairs tested.

For recording, slices were perfused with an extracellular solution as follows: 130 mm NaCl, 2.5 mm KCl, 1.25 mm NaH_2_PO_4_, 25 mm NaHCO_3_, 10 mm glucose, 2.5 mm CaCl_2,_ and 1.5 mm MgSO_4_, saturated with 95% O_2_/5% CO_2_, pH 7.4, at 33–35°C. Patch‐clamp recordings were made from neurons in L4 of the barrel cortex visualized using infrared illumination and differential interference contrast (DIC) optics. Whole‐cell recordings were made with patch electrodes (4–7 MΩ) filled with 135 mm cesium methane sulfonate, 8 mm NaCl, 10 mm HEPES, 0.5 mm EGTA, 0.5 mm Na‐GTP, 4 mm Mg‐ATP and 5 mm QX 314, pH 7.3, 290 mOsm. Biocytin (1 mg/ml) was added to whole‐cell solution in a subset of recordings. L4 contains both stellate cells and star pyramidal cells. If we preferentially recorded from different cell types in different genotypes this may bias our data. We filled a subset of cells during these principal cell recordings and found that we recorded a similar proportion of stellate cells and star pyramidal cells (referred to collectively as SCs) in both genotypes (WT 7/13 stellate, R43Q 6/13 stellate). For recordings in Figure 2 , a current clamp solution was used in which cesium methane sulfonate was replaced with potassium methane sulfonate and QX 314 was omitted. TC excitatory postsynaptic currents (EPSCs) were evoked at a frequency of 0.2 Hz by electrical stimulation of TC axons by a bipolar stimulating electrode placed in the ventrobasal thalamus. For EPSC recordings, in Figure 1 50 μm picrotoxin was included to block GABA_A_ receptors. For other recordings, EPSCs and feedforward inhibitory postsynaptic currents (IPSCs) were recorded at GABA_A_ and glutamate reversal potential, respectively, determined empirically in each cell. Feedforward interneurons were located by characteristic morphology and location under DIC optics: large bipolar cells on the border of L4 and layer 5 (L5).^25^ They were confirmed as fast‐spiking interneurons (FS) by examining their firing response to 500 msec current steps as outlined in results and previous work.^25^ During paired recordings, action potentials were evoked in SCs or FS cells by 1 msec current steps at 0.05 Hz. Local, pharmacologically isolated IPSCs in Figure 5 were evoked by placing the stimulating electrode on the border of cortical L4 and L5, close to the recording site, and stimulating at 0.2 Hz. Unitary EPSC and IPSC amplitudes were measured from an average of all trials; failures of transmission were not observed.

Recordings were made using a Multiclamp 700B (Molecular Devices). Signals were filtered at 4 kHz and digitized at 10 kHz (firing patterns filtered at 10 kHz and digitized at 40 kHz) using Signal 4 (CED). We did not correct for junction potential. Series resistance was 10–30MΩ.

### Dual minimal stimulation experiments

Stimulation intensity was set at that at which an EPSC was first seen in either cell. The average failure rate for the first cell to respond to TC stimulation (cell A) in these experiments is 0.57, and each experiment consisted of an average of 37 trials (range 25–62 trials). The same axon was deemed to contact both cells if the probability of producing the same or greater number of trials in which both cells displayed EPSCs was <0.05. This was tested using the binomial distribution versus the chance level of coincident successes, given the success rate in each cell. In experiments when the axon was deemed to contact both cells, the proportion of successes in cell A in which both cells responded = 0.95 ± 0.02, n = 15, range = 0.73–1 (1 for 9/15 experiments). In experiments when the same axon was not deemed to contact both cells, coincident successes = 0.02 ± 0.02 n = 20, range = 0–0.42 (0 for 18/20 experiments).

### Data analysis

All electrophysiologic parameters were determined in Signal 4 and decay kinetics were based on a double exponential fit. For 50 Hz stimulation experiments, IPSC charge (area under curve) was calculated 20–140 msec after the first stimulus corresponding to the time from second stimulus until the end of the resulting burst. We did not attempt to measure short‐term plasticity of IPSC amplitude during these trains, as individual, feedforward IPSCs were often impossible to isolate within a larger, compound IPSC from the second stimulus onward. Number of cells is denoted by n, whereas number of animals is denoted by N. Within‐animal averages were first calculated and all statistical analyses carried out between animals with the exception of correlation analysis, which requires the values from individual experiments. For these experiments, Pearson's correlation coefficients were calculated in SigmaPlot and correlation coefficients between genotypes were tested using the Fisher r to z transformation. Connectivity rates were tested using Barnard's exact test in Matlab. FS‐to‐SC EPSC ratio values were log‐transformed to produce a normal distribution before differences were tested with a *T*‐test. Log transformation did not result in a normal distribution of GABA‐to‐AMPA ratio values, but data were displayed as Log (ratio+1) for ease of viewing and data were tested with a Mann‐Whitney test. Unless otherwise stated, all other statistics were tested using two‐tailed unpaired *T*‐tests.

## Results

### Properties of TC synapses and TC connectivity

To determine if the properties of individual TC synapses are altered in R43Q heterozygous (referred to as R43Q) mice, we recorded from L4 stellate and star pyramidal cells (SCs) in TC slices taken from developing (P9–11) wild‐type (WT) and R43Q mice during minimal stimulation of the ventral posteromedial thalamus (Fig. 1A–H). Minimal stimulation intensity was set by gradually increasing intensity from zero until the lowest intensity at which EPSCs were seen in a proportion of trials (mean failure rate was 0.64 ± 0.03) as described previously.^29^ EPSCs evoked by minimal stimulation (msEPSCs) are generated by a single TC axon. We found that the amplitude of msEPSCs is not altered in R43Q mice (msEPSC: WT 34.3 ± 7.8 pA, N = 13, n = 24, R43Q 28.0 ± 4.0 pA, N = 10, n = 16, p = 0.51, Fig. 1A,B). The kinetics of msEPSCs are also unaltered in R43Q mice (10–90% rise time: WT 1.3 ± 0.3 msec, R43Q 1.2 ± 0.1 msec, p = 0.58, Fig. 1A,B; fast decay time constant WT 3.3 ± 0.3 msec, R43Q 3.0 ± 0.3 msec, p = 0.48, Fig. 1A).

We recently demonstrated an experience‐dependent increase in the proportion of L4 SCs contacted by each TC axon.^29^ This increase in connectivity is likely mediated by neuronal activity. Thus it would be expected that altered activity, resulting from a global decrease in GABAergic transmission mediated by γ2 receptors, may modify the increase in connectivity. To test this we recorded from 2 L4 SCs simultaneously during minimal thalamic stimulation (Fig. 1H). If an msEPSC is evoked in only one SC, this demonstrates that an individual TC axon makes a functional synapse onto only one of the two SCs (Fig. 1C,E). If msEPSCs are evoked simultaneously in both SCs this suggests that the same TC axon makes functional synapses onto both SCs (Fig. 1D,F). The proportion of experiments in which both SCs are contacted by the same TC axon can be used to estimate the proportion of SCs contacted by each TC axon.^29^ We found no difference in the proportion of SCs contacted by each TC axon in R43Q mice (proportion cells: WT 7/19 = 0.36, R43Q 8/16 = 0.5, p = 0.3, Fig. 1G), and the proportion of SCs contacted corresponds well to previously reported figures.^29^, ^30^

### Reduced local input to fast‐spiking interneurons

The developmental recruitment of feedforward inhibition^25^ in the TC circuit has also been shown to be experience‐dependent via an increase in the TC input to fast‐spiking interneurons (FS).^24^ We examined the relative TC input to FS cells by recording simultaneously FS cells and SCs during thalamic stimulation^25^ (Fig. 2A). Unlike msEPSCs, the absolute amplitude of EPSCs evoked by non‐minimal stimulation cannot be compared directly, as amplitude depends on factors such as stimulation intensity and exact angle of slice cutting. It is important, however, that equivalent amplitudes are examined in both genotypes, as the relationship between cell types may be different at different amplitudes. To assess this, we compared amplitudes between genotypes in this and all subsequent experiments featuring evoked TC EPSCs in SCs, and found no difference in amplitude (WT 112 ± 11 pA, N = 50 animals, R43Q 95 ± 11 pA, N = 67 animals, p = 0.147, two‐way analysis of variance [ANOVA]). FS cells were identified by narrow action potential (AP) (half width: 0.48 ± 0.02 msec, n = 38 cells), brief delay from peak of AP to trough of afterhyperpolarization (AHP) (delay: 0.82 ± 0.05 msec) fast, large amplitude AHP (amplitude: 22.9 ± 0.6 mV, half‐width: 23.3 ± 1.8 msec), and high frequency firing in response to current steps of double rheobase amplitude (APs per 500 msec: 43 ± 3, Fig. 2B) as reported previously.^25^ Firing properties did not differ between genotypes (data not shown). TC EPSCs evoked in FS cells were larger than in SCs in both WT and R43Q (WT SC EPSC: 70 ± 15 pA, FS EPSC: 235 ± 61 pA, N = 12, n = 12, p = 0.014; R43Q SC EPSC 44 ± 10 pA; FS EPSC: 165 ± 40 pA, N = 19, n = 20, p = 0.005, Fig. 2C,D), and the FS‐to‐SC ratio did not vary between genotype (WT log ratio 0.55 ± 0.17, R43Q: 0.54 ± 0.13, p = 0.97, Fig. 2E), demonstrating that TC input to FS cells is not altered by the *R43Q* mutation. The TC feedforward circuit also comprises inhibitory synapses between FS cells and SCs. The probability of connections between FS cells and SCs was not different between genotypes (WT: 7/17 = 0.41, R43Q: 6/18 = 0.33, p = 0.39, Fig. 2F,H). The amplitude of unitary IPSCs was also the same in WT and R43Q mice (WT: 34 ± 14 pA, N = 6, n = 7, R43Q: 39 ± 16 pA, N = 6, n = 6, p = 0.65, data not shown). During these recordings we also examined the recurrent connections made by SCs on to FS cells and, surprisingly, found a substantially lower connection probability in R43Q mice (WT: 10/17 = 0.59, R43Q: 3/20 = 0.15, p = 0.004, Fig. 2G,H). There was no difference in unitary EPSC amplitude (WT: 20 ± 10 pA, N = 10, n = 10, R43Q: 37 ± 26 pA, N = 3, n = 3, p = 0.44, data not shown), although the small number of unitary EPSCs resulting from this low connection probability in R43Q mice precluded a meaningful comparison of the efficacies of these connections.

### Inhibition evoked by TC stimulation is normal after single stimuli but altered during bursts

These findings showing normal TC input to, and normal output from, FS cells suggest that feedforward inhibition produced by single TC stimuli would be unaltered in R43Q mice.

**Figure 3:**
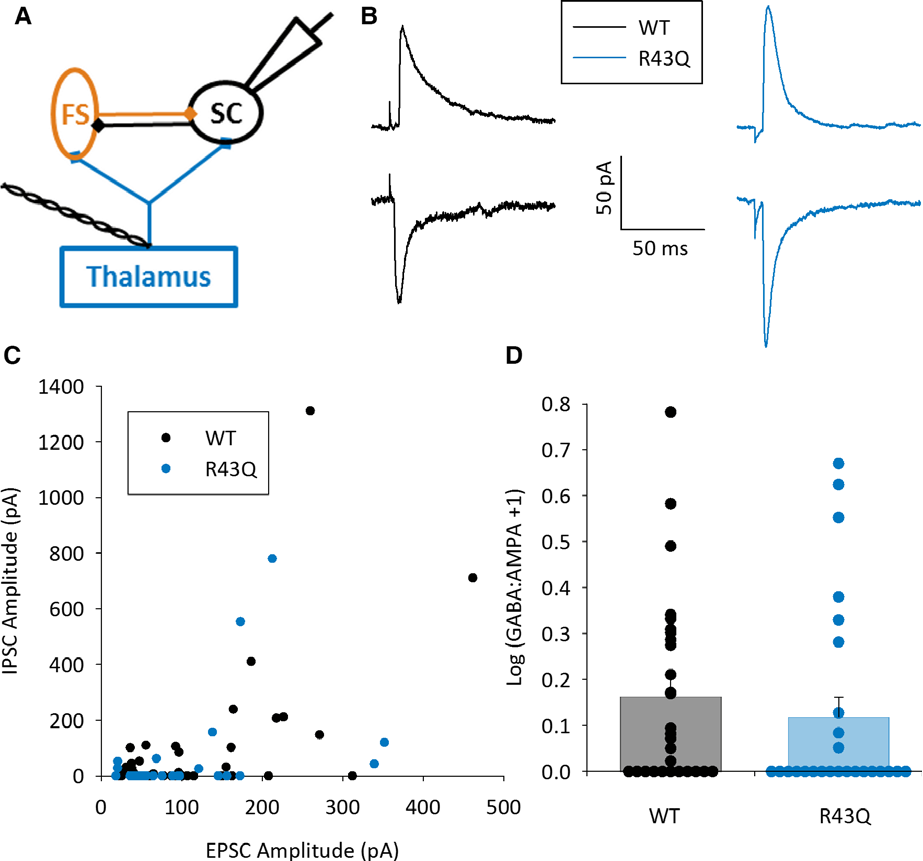
Normal feedforward inhibition in R43Q mice. (**A**) Schematic showing recording configuration SC in black, FS in orange, and thalamus and TC axons in blue. (**B**) Example traces showing TC EPSC recorded at GABA_A_ reversal potential (−60 mV, lower traces) and feedforward IPSC at glutamate reversal potential (+5 mV, upper traces) in WT and R43Q mice. (**C**) Graph showing mean EPSC versus IPSC amplitude for individual experiments in WT and R43Q mice. (**D**) Log GABA‐to‐AMPA ratio+1 for all experiments shown in **C**.

However, as well as modifying overall activity levels, GABA also acts as a trophic factor during development,^31^ and directly controls the migration of GABAergic interneurons from their birth in the ganglionic eminences to the cortex via GABA_A_ receptors.^32^ As such, altered surface expression of GABA_A_ receptors in R43Q mice may result in altered feedforward inhibition due to a change in the number of functional feedforward interneurons. To test this, we compared the peak amplitude of feedforward IPSCs to TC EPSCs in individual SCs in interleaved trials (Fig. 3A). Because we have already demonstrated that direct TC input to SCs is unaltered in R43Q mice, any change in the relative strength of inhibitory and excitatory inputs would reflect changes in the strength of feedforward inhibition. Our recordings showed, however, that the GABA‐to‐AMPA ratio was not altered by the presence of the R43Q point mutation (Log (GABA/AMPA + 1) WT: 0.16 ± 0.04, N = 28, n = 37, R43Q: 0.12 ± 0.04, N = 26, n = 26, p = 0.14, Fig. 3B–D), demonstrating that feedforward inhibition in response to single thalamic stimuli is unchanged.

**Figure 4:**
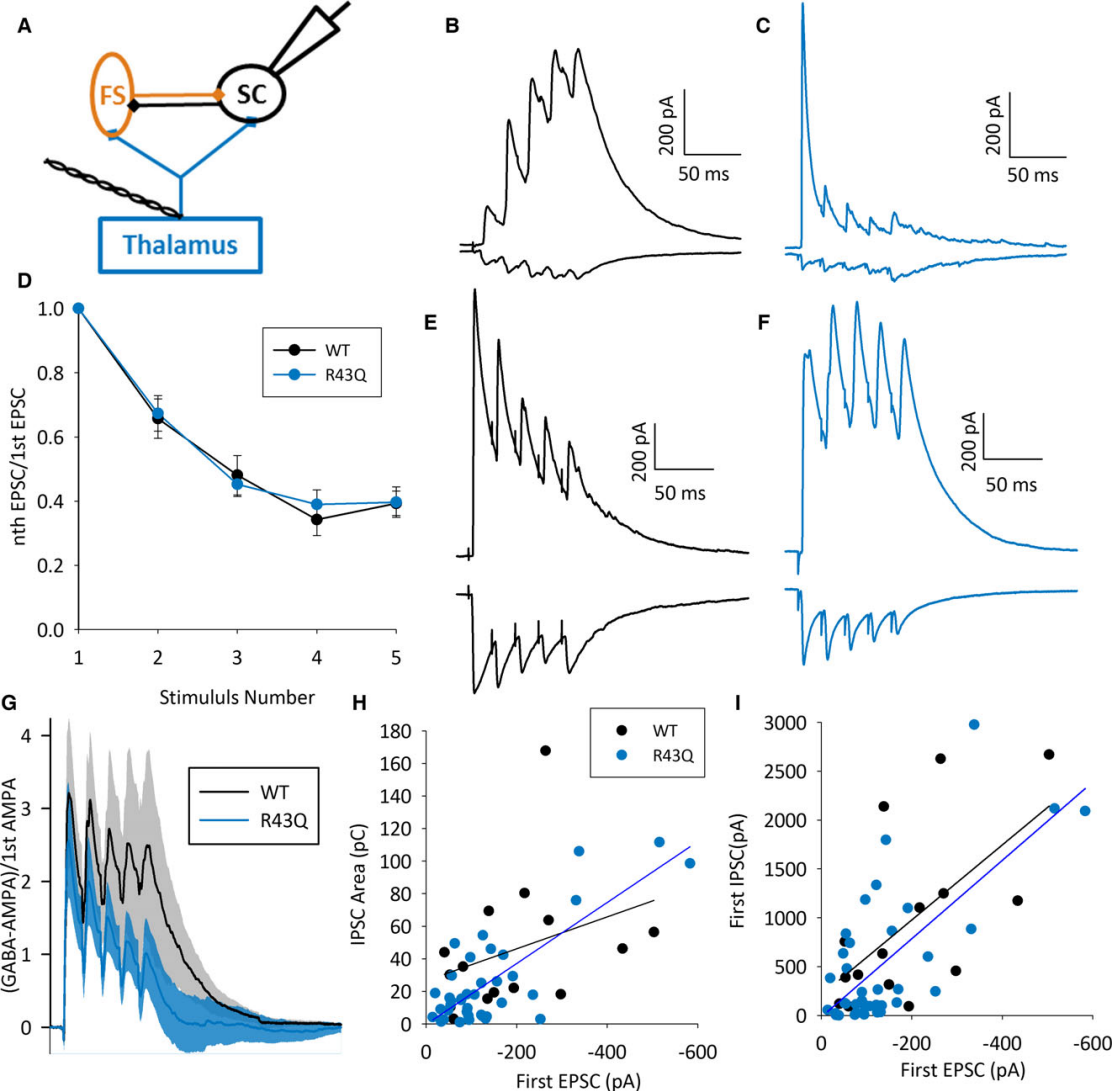
Altered inhibition during trains of thalamic stimulation. (**A**) Schematic showing recording configuration SC in black, FS in orange, and thalamus and TC axons in blue. (**B**) Example traces showing EPSCs recorded at −50 mV and IPSCs recorded at +10 mV during 50 Hz thalamic stimulation in an SC in a WT mouse. Traces show average of all sweeps. (**C**) As for **A** except in R43Q mouse. (**D**) Graph showing EPSC amplitude in response to 50 Hz thalamic stimulation normalized to the amplitude of the first EPSC. Black circles show average data for all experiments in WT mice including those with both small (as seen in **B** & **C**) and large (as seen in **E** & **F**) amplitude EPSCs; blue circles show average data for all experiments in R43Q mice. (**E**) As for **A** but showing WT example with large TC input. (**F**) As for B but showing R43Q example with large TC input. (**G**) Grand average across all 50 Hz stimulation experiments in WT (black) and R43Q (orange) mice. Traces show the sum of the traces recorded at GABA reversal potential and glutamate reversal potential (effectively GABA‐AMPA). This was calculated for the average of all sweeps at each potential within each experiment, averaged within each experimental animal, and then averaged across animals. Shaded areas represent the standard error between animals. (**H**) Scatter plot of peak amplitude of first EPSC versus IPSC charge (area under the curve) from 20–140 msec after first stimulus for all 50 Hz stimulation experiments. Solid lines show a linear fit. (**I**) Scatter plot of peak amplitude of first EPSC versus peak amplitude of first IPSC for all 50 Hz stimulation experiments. Solid lines show a linear fit.

TC cells recorded in vivo in developing mice fire bursts of action potentials at approximately 50 Hz in response to whisker stimulation.^33^ During this type of activity there is substantial firing of local SCs, so that recurrent excitation is likely to play a major role in the activation of FS cells. We hypothesized that, due to the reduced SC‐FS connectivity, inhibition recruited by bursts of 50 Hz thalamic firing would be reduced in R43Q mice. To test this, we recorded SCs at GABA and glutamate reversal potential during trains of 50 Hz thalamic stimulation. As expected, 50 Hz stimulation produced clear disynaptic, feedforward inhibition in response to the first stimulus with polysynaptic elements in response to subsequent stimuli (Fig. 4B,C,G). It is notable that we found that there is no difference in the short‐term plasticity at TC synapses onto SCs (p genotype effect = 0.87, two‐way ANOVA with repeated measures, Fig. 4D), so that a change in presynaptic function is unlikely to influence the response to 50 Hz stimulation. To test if this polysynaptic inhibition was reduced in R43Q mice, we calculated the balance of excitation and inhibition throughout the train by subtracting the current at GABA reversal potential from that at glutamate reversal potential and normalizing this to the amplitude of the first EPSC. Although inhibition appeared lower in response to stimuli 2–5 in R43Q mice (Fig. 4C,G), there was no significant difference between genotypes (inhibitory area 20–140 msec after first stimulus, WT: 233 ± 97, n = 15, N = 10, R43Q: 102 ± 43, n = 39, N = 21, p = 0.14, Fig. 4G). We noticed, however, that this trend for lower inhibition was most pronounced in cells from R43Q mice receiving relatively small TC EPSCs (compare Fig. 4B,C with E,F). Therefore, we examined the relationship between excitation and inhibition in individual cells and found that in WT cells the total charge produced during the train is not linearly correlated with the amplitude of the first EPSC (R = 0.34, p = 0.22, n = 15, Fig. 4B,E,H). In R43Q cells, however, there was a strong positive correlation between first EPSC amplitude and total IPSC charge (R = 0.80, p < 0.0001, n = 39, Fig. 4H), which was significantly different from the correlation in WT cells (p = 0.026). This difference was not a result of changes in feedforward inhibition as, similar to single stimulus experiments, the amplitude of the initial feedforward IPSC was tightly linked to that of the first EPSC in both genotypes (WT: R = 0.61, p = 0.015, n = 15, R43Q: R = 0.70, p < 0.0001, n = 39, Fig. 4I). These data indicate that, as a result of the reduced connectivity from SCs to FS cells, recurrent inhibition is only engaged by strong TC stimulation.

### Normal total levels of GABAergic inhibition in L4 stellate cells

**Figure 5:**
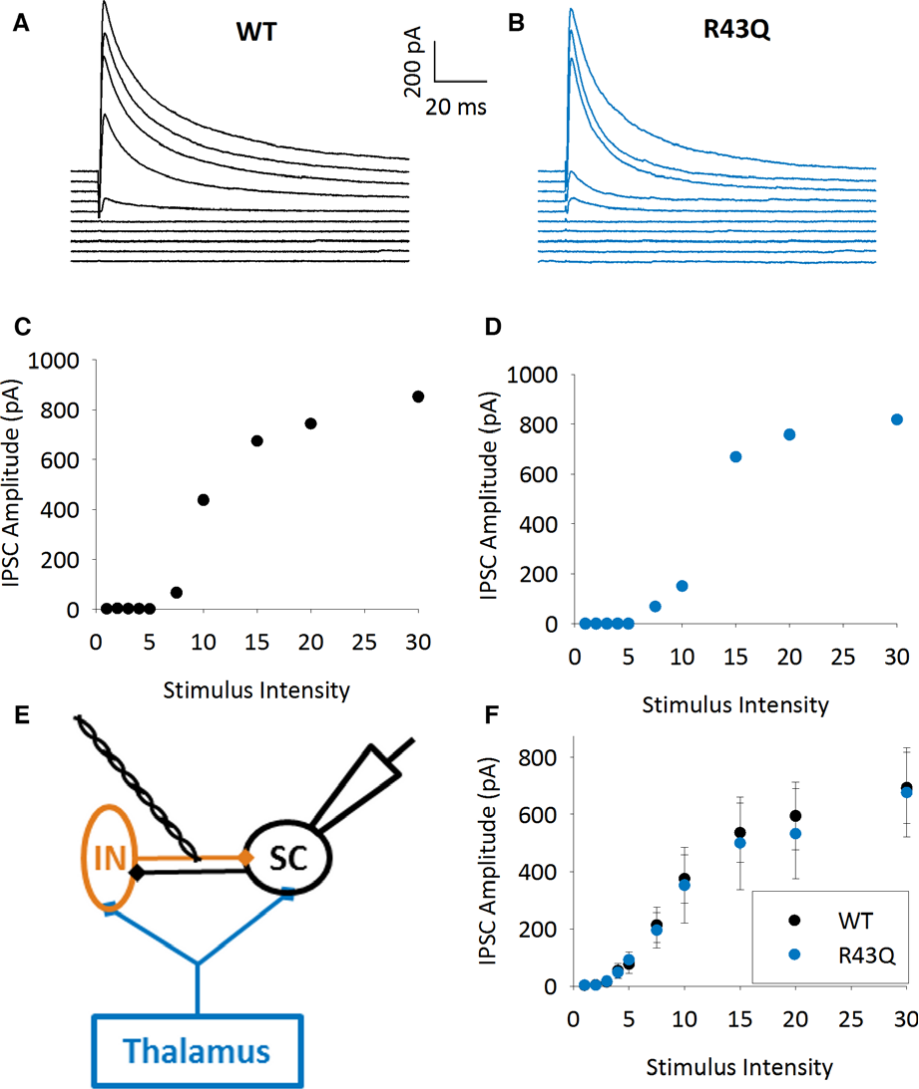
GABAergic IPSCs evoked by local stimulation. (**A**) Example traces showing IPSCs from SC evoked by local stimulation in the presence of NMDAR and AMPAR antagonists in a WT mouse. Traces show increasing stimulation intensity from 1 V (bottom trace), 2, 3, 4, 5, 7.5, 10, 15, 20 to 30 V (top trace). (**B)** As **A** but in R43Q mouse. (**C**) IPSC amplitude versus stimulus intensity plot for experiment shown in **A**. (**D**) IPSC amplitude versus stimulus intensity plot for experiment shown in **B**. (**E)** Schematic showing recording configuration SC in black, interneurons in orange, and thalamus and TC axons in blue. (**F**) Summary graph showing mean IPSC amplitude for all experiments as shown in **A**–**D**.

Feedforward inhibition is mediated selectively by fast‐spiking, parvalbumin‐containing interneurons,^25^, ^34^, ^35^ but other classes of interneuron mediate GABAergic cortical inhibition,^36^ which may also be affected by reduced levels of surface GABA_A_ receptors. Any changes in the level of inhibition produced by these other classes of interneuron may also contribute to the changes seen during 50 Hz stimulation. To test if the total level of inhibition impacting L4 SCs is altered in R43Q mice, we recorded from L4 SCs while stimulating GABAergic fibers locally in the presence of antagonists of glutamatergic transmission (50 μm D‐APV and 50 μm CNQX, Fig. 5E). Local axons were stimulated at a range of intensities from 1 V to 30 V, and the resulting IPSC recorded at 0 mV (Fig. 5A–F). There was no difference in the input‐output relationship or IPSC amplitude at any intensity between genotypes (IPSC at 30 V stimulation WT: 693 ± 124 pA, n = 9, R43Q: 676 ± 156 pA, n = 14, p > 0.05 two‐way ANOVA across stimulation intensities, Fig. 5B–F).

## Discussion

Pharmacologic inactivation of S1 blocks absence seizures in rodent models.^5^ The development of the TC input to S1 is highly sensitive to neuronal activity during a critical period in the first postnatal week in rodents. GABAergic signaling plays a vital role in cortical development including regulating critical periods.^27^ Given these important functions, the reduced surface expression of the near‐ubiquitous GABA_A_ γ2 subunit in R43Q mice would be expected to have profound effects during TC development. Any alterations in development could explain the protective effect of suppression of the R43Q allele during this early postnatal period.^21^ We find that, although the R43Q mutation has no effect on the direct TC input to either SCs or FS interneurons, the recurrent recruitment of inhibition during bursts of cortical activity is reduced in R43Q mice.

### Reduction in recurrent inhibition

The primary defect identified here in R43Q mice is a reduction in the local connectivity from L4 SCs to FS interneurons. We also find a change in the relationship between TC drive and inhibition during burst stimulation. In wild‐type mice, even weak TC stimulation results in strong inhibition throughout the duration of stimulation. In R43Q mice, however, similar inhibition is observed only during strong TC stimulation. Even though we found unchanged total inhibitory capacity in SCs, our findings suggest a reduced utilization of this capacity during bursts of cortical activity that may lead to hyperexcitability in vivo and potentially contributes to the seizure phenotype in these animals. Indeed, principal cells have been shown to be recruited to epileptiform activity only on cessation of the IPSC barrage that this activity produces.^37^ We think it unlikely, however, that this is the single cause of seizure generation, but rather may be a precursor to other defects such as the reduction in inhibitory drive on to layer 2/3 (L2/3) cells discussed below.^10^ In response to single stimuli, FS cells are likely activated largely directly by TC synapses and, as expected, there is no genotype difference in this initial feedforward inhibition. One scenario that could explain the difference between genotypes later in trains of TC stimuli is if, in wild‐type mice, the high level of local connectivity (59%) were to result in recurrent activation of FS cells in response to firing of even a small number of local SCs. In this situation, the majority of FS cells would be active even with weak TC stimulation, so stronger TC stimulation would have little impact. In R43Q mice, however, the high level of frequency‐dependent depression of TC synapses onto FS cells^35^ coupled with low local connectivity means that FS cells would be activated later in the train only if a high proportion of local SCs fired. Thus, stronger stimulation (larger first EPSC) would result in greater inhibition.

Although other classes of interneurons may be involved in inhibition during bursts, we find that the total level of inhibition in L4 is not altered, suggesting that the observed deficits in inhibition are unlikely to be a simple reduction in number of synaptic GABA_A_ receptors. Furthermore, the other classes of interneurons that are fully integrated in to the cortical circuitry at this developmental stage is not well characterized.^38^

How the γ2 *R43Q* mutation leads to a reduction in local connectivity to FS cells is not clear. Experience‐dependent activity alters the strength of TC input to FS cells,^24^ but we found no change in these synapses in R43Q mice. The effect of experience and activity on the development of local input to FS cells has not been tested, but any alteration in levels of activity caused by the *R43Q* mutation may be responsible. To our knowledge, local connectivity to cortical FS interneurons has not been studied previously in absence epilepsy models in either the somatosensory cortex or in other cortical regions. A reduction in AMPA receptor expression specific to parvalbumin interneurons has, however, been reported in stargazer mice,^39^ suggesting that reduction in the activation of cortical FS cells may be a common mechanism in absence‐like seizures. Changes in cortical inhibition do not rule out alterations in thalamic properties as a contributing factor to the generation of seizures, and it is possible that both cortical and thalamic mechanisms exist either within individual patients/animals or as alternative mechanisms resulting in the same seizure phenotype.^40^

### Total inhibition is unaffected in R43Q mice during early development

The finding that maximum local IPSC amplitude is unchanged is surprising given the reduction in inhibition in L2/3 of somatosensory cortex in R43Q mice.^10^ γ2 Subunits are thought to be included at all synaptic GABA_A_ receptors throughout development,^11^ so reduced surface expression would be expected to reduce cortical IPSCs. Although γ2 subunits are present in the cortex at this age, it is possible that the specific synapse types recorded in this study do not normally contain γ2 subunits, that other subunits compensate or that the mechanisms of GABA_A_ receptor synapse delivery in these neurons differ from that in L2/3 neurons so that there is no primary change in surface expression.

In summary, we have found a reduction in the recruitment of inhibition by bursts of cortical activity. This change occurs more than a week before the appearance of seizures on EEG. This finding reinforces the concept that the root cause of neurodevelopmental disorders may significantly precede the onset of noticeable symptoms.

## Disclosure

None of the authors has any conflict of interest to disclose. We confirm that we have read the Journal's position on issues involved in ethical publication and affirm that this report is consistent with those guidelines.
